# An expert-informed framework of school conditions potentially supporting adolescent physical activity habits: a three-stage consultation in China

**DOI:** 10.3389/fpubh.2026.1962254

**Published:** 2026-10-09

**Authors:** Shaojuan Hu, Yi Su, Dong Han, Liting Li, Ran Wang, Yihang Liu, Jie Xiong, Xinyuan Li

**Affiliations:** 1 College of Physical Education and Sports Science, Hengyang Normal University, Hengyang, China; 2 Shenzhen No. 3 Senior High School, Shenzhen, China

**Keywords:** adolescent, China, expert consultation, habit support, modified Delphi approach, physical activity, school health promotion

## Abstract

**Background:**

Schools can provide conditions that may support repeated physical activity, but expert endorsement of those conditions does not demonstrate habit formation. This study developed an expert-informed content framework for Chinese adolescents aged 12–18 years.

**Methods:**

A three-stage, Delphi-informed consultation comprised qualitative elicitation and item development, one full quantitative rating stage, and targeted review of proposed final decisions. Of 25 experts approached, 23, 18, and 16 were reported to complete Stages 1, 2, and 3, respectively. The reported analyses used 16 complete cases. Seven earlier-stage contributors withdrew consent; their data are excluded and recorded as no longer retained, preventing full-stage reanalysis. Five theory-informed domains guided the interviews; a predominantly deductive qualitative analysis using a codebook specified before the interviews supported indicator refinement rather than independently establishing their structure. Forty-five literature-derived provisional indicators and five interview-derived additions were consolidated into 34 candidates. Importance, feasibility, and clarity were rated on five-point scales. The importance-based retention rule specified before rating combined a mean ≥4.00, coefficient of variation ≤0.20, and ≥80% ratings of 4 or 5.

**Results:**

Thirty-three candidates met the retention rule; SPC9 did not. The retained indicators received uniformly high importance endorsement, but concordance in relative rankings was limited (Kendall’s W: importance 0.115, feasibility 0.160, clarity 0.163). Individual-level Stage 3 confirmation distributions were not available in the submitted analytic record, and quantitative convergence across stages was not assessed. The exploratory, sample-relative importance–feasibility matrix assigned 10, 5, 10, and 8 indicators to its four quadrants; 11 of 33 indicators changed quadrant in at least one leave-one-expert-out analysis.

**Conclusion:**

The resulting curricular, extracurricular, peer, cultural, and institutional indicators specify school conditions that experts considered potentially relevant to habit support. The findings do not establish strong inter-expert consensus, a validated measurement instrument, actual habit formation, or causal effects. Complete-case selection, limited stakeholder representation, and incomplete process records restrict interpretation. More complete source documentation, stakeholder co-design, and prospective measurement and implementation studies are needed.

## 1 Introduction

Insufficient physical activity during adolescence remains a persistent public health problem. The World Health Organization recommends that children and adolescents accumulate an average of at least 60 min per day of moderate-to-vigorous physical activity across the week, with vigorous and muscle- and bone-strengthening activity on at least 3 days (1). Global surveillance nevertheless indicates that about four in five school-going adolescents do not meet recommended levels, with a particularly marked disadvantage among girls (2). Cross-national report-card evidence likewise shows that overall physical activity and supportive policy implementation remain weak in many countries (3). Activity also tends to decline across adolescence, when academic demands, changing social priorities, and reduced access to structured play converge (4). In China, national consensus recommendations and policy reviews similarly identify low participation, uneven access, and a persistent gap between policy intent and school-level implementation (5, 6).

The public health relevance of physical activity extends beyond physical fitness and cardiometabolic risk. Objectively measured activity is favorably associated with physical, cognitive, and psychosocial health indicators in school-aged populations (7). Higher physical activity and lower sedentary behavior are also associated with better mental health, although effect sizes and causal certainty vary across outcomes and study designs (8). A meta-analysis found a small reduction in depressive symptoms immediately after physical activity interventions, but the limited follow-up evidence did not show a sustained advantage (9). Increasing activity during program delivery and maintaining activity after external support ends should therefore be treated as related but distinct objectives.

School-based interventions are well positioned to address the first problem because schools reach adolescents repeatedly and at scale. Their impact on whole-day physical activity, however, is often small or inconsistent. Reviews using device-measured outcomes have reported modest effects, substantial heterogeneity, and limited evidence of maintenance (10, 11). The school environment—policy, facilities, social climate, organized opportunities, and staff practice—can either enable or constrain activity, but no single environmental feature is sufficient (12). A recent secondary-school review found that promising programs commonly combined additional activity sessions, environmental changes, teacher training, peer support, and educational resources, while pooled effects on device-measured activity remained small or non-significant (13). Implementation reviews further show that intervention effects depend on staff capacity, organizational fit, leadership, resources, and the fidelity–adaptation balance (14, 15). Likewise, comprehensive school physical activity programs are conceptually attractive, yet their components, implementation quality, and reported outcomes vary considerably (16–18).

These findings support a whole-school perspective but leave a practical question unresolved: what should schools organize if the objective is not only more activity during a program, but a pattern of physical activity that students are increasingly able and willing to sustain? Health-promoting school standards and comprehensive school physical activity models establish a valuable settings-based foundation (17–20). Most were not designed, however, to specify the behavioral conditions under which repeated physical activity becomes context-linked and less dependent on moment-to-moment deliberation. A framework for habit formation therefore needs to preserve the ecological breadth of whole-school models while making the proposed behavioral pathway more explicit.

Three bodies of theory informed the present study. First, social-ecological models locate adolescent behavior within interacting individual, interpersonal, organizational, community, and policy environments (21). Schools are not a single exposure in this account; they are organized systems in which curricula, schedules, peer relations, norms, resources, and rules operate together. Bronfenbrenner’s ecological account further emphasizes that development reflects recurring interactions between a young person and the settings in which everyday life unfolds (22). Repeated school routines are therefore not merely a backdrop to behavior. They can structure when activity is possible, who participates, and whether participation is reinforced.

Second, self-determination theory helps explain why the same opportunity can be engaging for one student and alienating for another. Environments that support autonomy, competence, and relatedness are more likely to foster self-determined motivation and sustained engagement (23). In physical activity research, perceived competence, enjoyment, and more autonomous regulation are consistently associated with participation, including in child and adolescent samples, although the evidence is stronger for association than for any single causal pathway (24, 25). A school framework concerned with maintenance should therefore attend not only to the supply of activities, but also to differentiated challenge, successful experiences, meaningful choice, non-exclusion, and supportive social relationships.

Third, habit theory distinguishes repeated behavior from habit as an acquired cue–response association. When a behavior is repeated in a sufficiently stable context, contextual cues can begin to trigger action with less deliberative effort (26). The time required varies widely across people and behaviors; habit formation is not a fixed “number of days” (27, 28). Habit also does not make motivation irrelevant. Instead, reflective motivation can help initiate and repeat behavior, whereas contextual stability and repetition may gradually make enactment more efficient (29, 30). Longitudinal evidence generally supports a positive relationship between physical activity habit strength and behavior, but directionality and measurement remain heterogeneous (31). Measurement studies likewise caution against treating frequency as equivalent to automaticity (32, 33). Habit-focused interventions can strengthen physical activity habits, although much of the evidence comes from adults and short follow-up periods (34). For adolescents, protected times, recurring activity formats, familiar partners, and feedback may be plausible habit-supportive conditions, but this proposition requires empirical testing.

Peer relations are especially salient during adolescence. Friends can influence activity through co-participation, invitations, norms, acceptance, encouragement, and, negatively, exclusion or victimization (35). Social support is positively associated with adolescent girls’ physical activity, but its effects depend on the source and form of support (36). Reviews of peer-led strategies report promising elements but heterogeneous intervention designs and outcomes (37). Chinese panel evidence also suggests that classmates’ activity and the class sport atmosphere are associated with individual participation, while underscoring the difficulty of isolating peer causality (38). These findings argue against treating peer support as a generic background factor. A school framework should specify observable peer processes that can be designed or monitored.

Recent Chinese studies have developed broader frameworks for youth physical activity promotion. Kong et al. (39) used Delphi and analytic hierarchy process methods to organize responsibilities across school, family, community, and societal domains. Luo and Chen (40) subsequently proposed and empirically applied a four-domain evaluation system of government, school, family, and community support. Policy-history research shows that national promotion efforts have progressively expanded while implementation challenges have persisted (6). Those contributions address cross-sector governance and weighting. The present study asks a narrower question: within the school setting, which curricular, extracurricular, peer, cultural, and institutional conditions are considered important for adolescent physical activity habit formation? This within-school focus is intended to add behavioral and implementation detail rather than duplicate a broad socio-ecological map.

This study aimed to develop an expert-informed content framework describing school conditions that may support adolescent physical activity habits. The objectives were to (1) assemble and refine a pool of school-level indicators; (2) describe expert ratings of their importance, feasibility, and clarity; (3) make the operational boundaries of potentially overlapping indicators explicit; and (4) propose theoretical links for subsequent testing. The study did not measure student habit strength, longitudinal physical activity change, or causal pathways. It also did not assess factor structure, reliability, construct or predictive validity, responsiveness, or measurement invariance.

## 2 Materials and methods

### 2.1 Study design and reporting approach

The study used a three-stage expert consultation informed by the modified Delphi approach, conducted between 25 December 2025 and 1 July 2026. The corrected participant register records Stage 1 from 25 December 2025 to 1 March 2026, Stage 2 from 15 March to 20 April 2026, and Stage 3 from 1 May to 1 July 2026. The stages comprised qualitative elicitation and item development, one full quantitative rating stage, and targeted review of proposed final decisions. They were labelled Rounds 1–3 in the submitted version; “stage” is used here to distinguish their different functions. Reported participation was 23, 18, and 16 experts, respectively, and the submitted analyses were restricted to 16 complete cases. Only Stage 2 generated a full quantitative candidate-rating matrix used in this report. Stage 3 was not analyzed as an iterative rerating stage, and between-stage convergence or stability of quantitative judgments was not assessed. The later qualitative analysis documented the relationship between interview material and item decisions; it was not an additional consultation stage. CREDES and other Delphi guidance inform the interpretation of these adaptations (41–45). Figure 1 distinguishes participation, analysis sets, and the limits of Stage 3 documentation.

**Figure 1:**
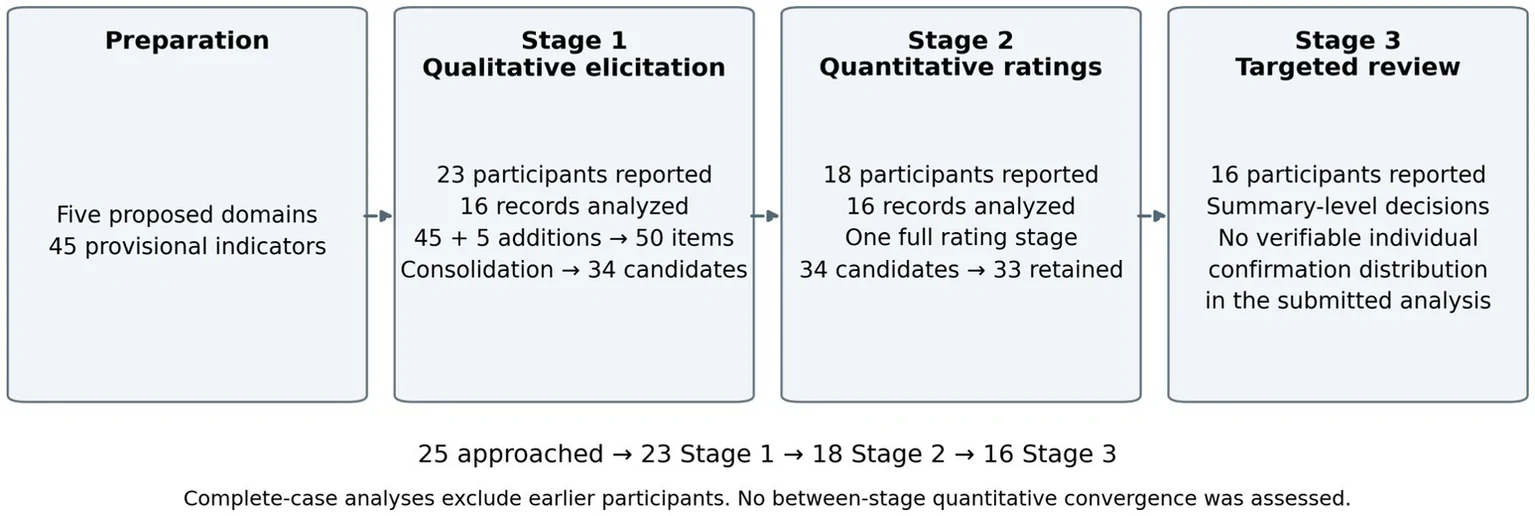
Three-stage consultation and reported analysis sets. The 25 → 23 → 18 → 16 participation flow is distinguished from the 16-expert complete-case analyses. Only Stage 2 contributes full quantitative ratings to the reported analyses. Stage 3 contributes a summary of decisions, not a verifiable individual confirmation distribution or evidence of increasing consensus.

The approach departed from a conventional iterative Delphi survey in two important respects: the first stage used theory-informed domain-specific interviews rather than independent quantitative ratings, and the final stage was a targeted review rather than full quantitative rerating. The five-domain starting structure also constrained the scope of elicitation. Completion of the planned stages is not evidence of convergence, unanimity, or saturation. Accordingly, the findings are described as expert endorsement and framework development, not as strong consensus established through three quantitative Delphi rounds. The revision also draws on ACCORD reporting guidance to make selection, stage objectives, feedback, missing records, and unresolved limitations explicit; this does not establish that all checklist requirements have been met (46).

### 2.2 Conceptual scope

For this study, adolescents were defined as students aged 12–18 years. School-based physical activity support was defined as the set of curricular, extracurricular, interpersonal, cultural, and organizational conditions through which a school enables adolescents to participate in physical activity repeatedly and with increasing competence, confidence, social acceptance, and continuity. Physical activity habit formation was treated as a proposed downstream process involving repeated initiation in recurring contexts and increasing automaticity. The framework was therefore designed around modifiable school conditions, not around student habit symptoms or health outcomes.

The provisional domains were: curricular support; extracurricular activity support; peer support; school physical activity culture; and institutional support. Curricular support concerned what students learn and experience in physical education. Extracurricular support concerned recurring opportunities beyond formal lessons. Peer support concerned invitation, encouragement, acceptance, and group participation. Culture concerned shared meanings and inclusive norms. Institutional support concerned time protection, resources, quality assurance, safety, staff development, coordination, and feedback. The social-ecological model provided the system structure (21); self-determination theory informed competence, autonomy, and relatedness processes (23, 24); and habit theory informed contextual stability and repetition (26, 27, 29, 31, 34).

### 2.3 Expert panel

Twenty-five experts were approached, of whom 23, 18, and 16 were reported to complete Stages 1, 2, and 3, respectively. Stage-specific completion was 92.0% (23/25), 78.3% (18/23), and 88.9% (16/18). The 16-person complete-case analysis set represented 64.0% of those approached and 69.6% of Stage 1 participants. The ethics application submitted as Data Sheet 1.PDF specifies long-term involvement in school physical education, curriculum and teaching, sport psychology, child and adolescent health promotion, or school physical education management; a senior professional title; at least 10 years of relevant work experience; familiarity with adolescent physical activity promotion or health-promoting schools; and voluntary informed participation. It also identifies willingness and ability to complete the consultation stages. The application describes recruitment from university physical education colleges, child and adolescent health research institutions, primary or secondary school physical education management, and regional teaching-research departments. These are the documented eligibility requirements and intended recruitment sources; they are distinct from verified characteristics of individual respondents. Experts were recruited purposively through professional referrals from institutional leaders and academic mentors. The researcher contacted the recommended experts individually and invited them to participate in the consultation. Recommendations provided the route of access to potential participants; they do not, by themselves, verify every eligibility criterion or establish representativeness.

Table 1 describes the 16-person analysis set. Recorded working experience ranged from 14 to 43 years (mean 26.69, sample SD 8.80; median 23.0). The revised register separately reports relevant-field experience of 10–32 years (mean 18.81, sample SD 6.82; median 16.5). All 16 included experts meet the stated minimum of 10 years of relevant experience. Yi Su (recorded as SY in the participant register) is identified as the qualifications checker for all 16 experts. Their recorded invitation dates range from 20 November to 5 December 2025 and denote invitations, not formal study consent. The updated register identifies 13 professors (81.3%), one researcher (6.3%), one associate researcher (6.3%), and one secondary-school participant with a senior professional title (6.3%). These are reported professional titles; researcher and associate researcher denote Chinese professional-title categories. All 16 titles are now specified. For two experts, the register records a research field without naming the institution. The primary-expertise categories are school physical education/curriculum/governance (7), exercise psychology/mental health (4), school or adolescent health promotion/health ecology (3), and exercise physiology/training (2). The municipal teaching-research and secondary-school management participants provide some school-practice representation. Part I of the full interview guide asks about expertise, relevant experience, professional activities, and familiarity. Initial acceptance of an invitation and informed consent at interview are distinguished. The interview and consent procedures are described in Sections 2.4 and 2.9. An expert authority coefficient was not calculated because the required source variables were unavailable.

**Table 1:** Characteristics of the complete-case expert panel included in the analysis (*N* = 16).

**Characteristic**	**Category**	**Value**
Analytic panel	E01–E16; completed all three stages	16
Recruitment flow	Approached → Stage 1 → Stage 2 → Stage 3	25 → 23 → 18 → 16
Complete-case retention	Of all experts initially approached	16/25 (64.0%)
Complete-case retention	Of Stage 1 participants	16/23 (69.6%)
Primary expertise	School physical education, curriculum, and governance	7 (43.8%)
Primary expertise	Exercise psychology and mental health	4 (25.0%)
Primary expertise	School/adolescent health promotion and health ecology	3 (18.8%)
Primary expertise	Exercise physiology and physical training	2 (12.5%)
Geographic coverage	Provinces/municipalities represented by affiliations	Beijing; Shanghai; Guangdong; Guangxi; Jiangxi; Jiangsu; Hunan
Relevant-field experience	Mean (sample SD), years	18.81 (6.82)
Relevant-field experience	Median; minimum–maximum, years	16.5; 10–32
Relevant-field experience	At least 10 relevant years	16 (100.0%)
Recorded working years	Mean (sample SD), years	26.69 (8.80)
Recorded working years	Median; minimum–maximum, years	23.0; 14–43
Practice-facing affiliation	Municipal educational research institute / physical education teaching-research	1 (6.3%)
Practice-facing affiliation	Secondary school/school-management focus	1 (6.3%)
Professional title	Professor	13 (81.3%)
Professional title	Researcher	1 (6.3%)
Professional title	Associate researcher	1 (6.3%)
Professional title	Senior professional title; secondary-school participant	1 (6.3%)

*N* = 16 denotes the reported complete-case analysis set. SD is the sample standard deviation. Relevant-field experience and total working years are separately reported from the updated register. All 16 included experts have at least 10 reported relevant years. All professional titles are taken from the updated author-supplied register; researcher and associate researcher denote Chinese professional titles. Percentages are rounded independently and may not sum to exactly 100%. Primary-expertise categories follow the submitted Table 1. No individual profiles of withdrawn experts are reported. None of the 16 included experts withdrew consent.

### 2.4 Stage 1: Expert interviews and item development

Stage 1 consisted of individual semi-structured interviews to refine the initial indicator pool. The administered Chinese guide contains expert-background questions, 32 numbered questions (Q1–Q32), and supporting prompts. It covers core concepts, five proposed domains, mechanisms, school implementation, measurement, prioritization, and refinement. The authors supplied the Chinese guide during revision and confirmed it as the guide used; an English version is provided in the Supplementary Interview Guide. The open-ended interview component used these 32 questions, rather than four questions. The guide explicitly presents domains, examples, and prompts, so the elicitation was theory-informed and prompted. Interviews in Hunan Province were conducted face to face and audio-recorded; experts outside Hunan participated through WeChat and/or telephone, with communication records retained. Jie Xiong and Liting Li transcribed the interview recordings. Ran Wang and Yihang Liu performed transcript text entry and checked and corrected transcription errors; Yi Su and Dong Han checked transcript accuracy. The excerpts in Supplementary Table S7 are English translations of experts’ original Chinese statements, not summaries written after the interviews. Researchers knew identities during recruitment and interviewing, and feedback between stages was anonymized. Supplementary Table S5 is a later question-level summary and is distinct from the administered guide. Written informed consent was obtained at each participating expert’s initial interview.

The exploratory qualitative analysis included E01–E16, the 16 experts who completed all three stages. The reported dataset contains 512 question-level responses to 32 interview questions and 436 meaning units. The ethics application lists inability to complete all three consultation stages as an exclusion criterion. The seven other Stage 1 participants withdrew consent, and their prior interview data are recorded as no longer retained or permitted for use. Six of these seven also signed additional forms in July 2026, without restoring permission to use their data. Their interview data remain excluded, and no interview content was reconstructed. The qualitative analysis describes the retained complete-case subset; its coverage may differ from that of all 23 original interview participants.

The five support domains and the 14-code analytical framework were specified before the interviews. The codebook remained unchanged during the study. The qualitative analysis was predominantly deductive and used the predefined categories to organize experts’ statements and refine indicator content. It did not independently derive or validate the five-domain structure. The codebook covered conceptual boundaries (C01–C04), the five proposed domains (C05–C09), contextual cues and repetition, motivation, and self-efficacy (C10–C11), and implementation, measurement, and indicator-review issues (C12–C14). Each code specified inclusion criteria, an exclusion boundary, and one primary code per response unit (Supplementary Table S6) (47). NVivo 14 for Windows (Lumivero) was used to support coding. The codebook preceded Stage 2 rating and was not revised in response to the quantitative results.

Qualitative findings were used to describe the relevance and refinement of proposed content, not to establish the number of domains, confirm a latent structure, or quantify Delphi consensus. Mention counts and proportions describe salience within the included interviews. The submitted account states that Shaojuan Hu and Xinyuan Li coded independently and Yi Su adjudicated unresolved disagreements. Separate pre-reconciliation coding files were not available for verification; consequently, neither percentage agreement nor Cohen’s κ can be estimated, and the independence and consistency of the original coding cannot be independently audited. A proportion-based value previously labelled “CVR (Lawshe)” in a working file was only 2p − 1, where p was an interview mention proportion. Since experts did not make the essential/useful-but-not-essential/not-necessary judgments required for formal CVR, this value is not a content validity ratio or a retention criterion (48). Mention proportions are not presented as a content validity index either.

Item development began with 45 provisional indicators extracted from the literature: nine curricular, nine extracurricular, nine peer, eight cultural, and 10 institutional indicators. The interviews generated five additions: guidance on appropriate activity load, safety, and recovery (CS10); reliable extracurricular access to facilities and equipment (EAS10); class- or signature-program physical activity culture building (SPC9); student physical activity records and feedback (IS11); and coordination among physical education, homeroom teaching, and mental health education (IS12). The resulting 50-item working pool was reviewed for conceptual relevance, redundancy, clarity, and consistency with the school-action boundary. Indicators were retained, revised, merged, removed, or added, producing 34 Stage 2 candidates: seven curricular, seven extracurricular, six peer, six cultural, and eight institutional indicators (Table 2). Supplementary Table S3 retains the source codes and maps them to the Stage 2 codes. The suffix M identifies an item merged before rating, and R identifies a substantive wording revision before rating. Based on item content and sequence, the duplicated PS8M header in the working file was assigned the Stage 2 code SPC1R.

**Table 2:** Item development and Delphi decisions.

Stage	Input/Participation	Decision process	Output
Preparation	45 theory- and literature-informed provisional indicators	Five-domain conceptual scoping	45-item provisional pool
Stage 1 qualitative elicitation	25 approached; 23 completed; 16 complete-case records analyzed (512 question-level responses; 436 substantive response units)	Complete-case qualitative analysis and item consolidation based on relevance, overlap, clarity, and the school-action boundary	45 provisional indicators + 5 interview-derived additions = 50-item working pool → 34 Stage 2 candidates
Stage 2 direct rating	23 eligible; 18 completed; 16 complete-case experts analyzed; 34 candidates rated for importance, feasibility, and clarity	Reported importance rule plus diagnostic feasibility/clarity review	33 met all criteria; SPC9 failed
Stage 3 targeted confirmation	18 eligible; 16 participants reported; summary-level decisions only	Targeted review described in the submitted account; no individual confirmation distribution	Summary records SPC9 exclusion, unchanged 33-item set, and closure

Reported participation was 25 approached → 23 Stage 1 → 18 Stage 2 → 16 Stage 3. Analyses use E01–E16. Seven excluded Stage 1 participants, including the two excluded Stage 2 completers, withdrew consent; their prior data are recorded as no longer retained or permitted for use. Additional July signatures did not restore permission to use those data (Supplementary Table S8). The 436 qualitative units are from 512 responses in the retained subset. Stage 2 supplies quantitative endorsement; Stage 3 supplies summary-level process documentation.

The 45 provisional indicators were extracted from the literature and organized within the proposed five-domain framework. Supplementary Table S3 traces these indicators and the five interview additions into the rated candidates. During revision, a targeted search completed on 12 September 2026 identified 22 publications and official documents published before the study. Supplementary Table S12 maps each original indicator to relevant supporting content, its location, evidence type and limitations; Supplementary Table S13 provides full references, DOI or official links, and the scope of verification. This retrospectively assembled mapping clarifies conceptual, policy, pedagogical and empirical support. It does not establish that each listed source was consulted during initial item generation. The contemporaneous item-level extraction log was unavailable, and no reproducible systematic-review procedure is claimed. Some links are indirect, some interventions combine several components, and a peer-led trial had a null primary result. The mapped evidence does not validate the 45 items, the later 33-item framework, or causal effects on habit formation.

### 2.5 Stage 2: Importance, feasibility, and clarity ratings

Eighteen experts completed Stage 2, and the analysis includes E01–E16. The ethics application lists inability to complete all three stages as an exclusion criterion. The consent template provides for destruction and non-use of prior data after withdrawal. E22 and E23 completed Stage 2 but did not complete Stage 3 and withdrew consent. Their July 2026 additional signatures did not restore data-use permission. Their original ratings are recorded as no longer retained and are excluded from use, so an 18-person reanalysis cannot be performed. No ratings were reconstructed or imputed. This restriction does not remove possible selection bias. The 16-person matrix contains importance, feasibility, and clarity ratings for 34 candidates on five-point scales. Importance denotes perceived relevance; feasibility denotes perceived implementation under ordinary school conditions; clarity denotes conceptual and linguistic comprehensibility. The reported anchors were 1 (very unimportant/infeasible/unclear), 2 (unimportant/relatively infeasible/relatively unclear), 3 (moderate), 4 (important/feasible/clear), and 5 (very important/feasible/clear).

The rating form presented each candidate’s code, domain, label, and operational description and allowed additional comments on wording or implementation. Aggregate statistics and de-identified comments informed item retention and the Stage 3 confirmation. Importance was the basis for retention; feasibility and clarity were used to identify implementation barriers and potential wording problems.

### 2.6 Operational retention rule and statistical analysis

Item summaries comprised the mean, sample SD (denominator n − 1), median, interquartile range, observed range, coefficient of variation (CV=SD/mean), and percentage rated 4 or 5. Before Stage 2 rating, the research team specified the retention rule: mean importance ≥4.00, importance CV ≤ 0.20, and ≥80% ratings of 4 or 5. At N = 16, the percentage rule requires at least 13 endorsements (81.25%). These are operational selection thresholds, not validated boundaries between essential and nonessential content. Mean 4 corresponds to the “important” category, the percentage rule requires broad endorsement, and CV was intended to limit dispersion relative to the mean. No universal Delphi standard validates this exact combination (44, 45). Because the ratings are ordinal and lack a meaningful ratio-scale zero, mean and CV are pragmatic descriptors; medians and rating distributions are necessary for interpretation. Feasibility and clarity were diagnostic rather than automatic deletion criteria, so important but difficult-to-implement supports could remain visible.

In this report, endorsement means a score of 4 or 5 on importance; relative-rank concordance means the extent to which experts order candidates similarly, summarized by Kendall’s W; and consensus refers to a broader process claim that would require an explicit definition and verifiable evidence. Meeting the operational retention rule is therefore reported as criterion-based endorsement, not proof of strong consensus. A *post hoc* threshold sensitivity analysis used the reported 16-person item summaries to describe how retention depends on the cutoffs (Supplementary Table S11). It did not replace the original rule, add participants, or assess attrition bias.

Kendall’s coefficient of concordance (W) was calculated separately for importance, feasibility, and clarity across the 34 Stage 2 candidates. Because the five-point ratings contained tied ranks, W was derived from the tie-adjusted Friedman statistic: W = *χ*^2^/[m(k − 1)], where m is the number of experts and k is the number of items. Values closer to 1 indicate greater concordance in relative rankings. Statistical significance was interpreted together with the magnitude of W and was not taken to indicate strong agreement (49). Analyses were conducted in Python 3.12 using NumPy and SciPy.

An exploratory importance-feasibility analysis was conducted after item selection using the unchanged Stage 2 scores of the 33 retained indicators. The retained-subset grand means defined the sample-relative crosshairs (importance 4.462; feasibility 3.977), and values at or above a crosshair were classified as relatively high. The four quadrants were labelled priority action, strategic challenge, support and monitor, and foundational investment. Sensitivity was examined in 16 leave-one-expert-out analyses, with item means and both crosshairs recalculated after each omission. The revised figure places indicators with identical coordinates at one labelled point; no coordinate displacement is used for classification.

### 2.7 Stage 3: targeted confirmation

Sixteen of the 18 Stage 2 participants were reported to complete Stage 3. The research-process section of the ethics application describes returning Stage 2 summaries, anonymized comments, and proposed revisions for final confirmation. The submitted consent form likewise distinguishes a second-stage rating questionnaire from third-stage confirmation. Supplementary Table S4 records proposed exclusion of SPC9, retention of the other 33 items without wording changes, and the stopping decision. It explicitly states that individual response distributions are unavailable in that summary and that Stage 3 was targeted confirmation rather than full rerating. These documents support the described purpose of Stage 3 but do not supply individual votes, confirmation percentages, or independently auditable evidence of unanimity. The summary is therefore process documentation rather than an additional quantitative assessment of consensus. Absence of a recorded objection is not equivalent to agreement by every participant.

The wording and codes of the 33 retained items did not change after Stage 2. Two analysis sets are therefore reported: all 34 candidates for retention decisions and concordance, and the 33 retained items for descriptive domain summaries and the exploratory implementation matrix. Stage 2 scores should not be applied to an item that is subsequently reworded substantively; such an item would require renewed assessment of clarity and content.

### 2.8 Conceptual mapping

Following item selection, the research team organized the 33 indicators according to their proposed primary function and linked the five domains to competence and self-efficacy, autonomous motivation and enjoyment, stable contextual cues and repetition, peer belonging and social reinforcement, and organizational continuity. This conceptual mapping was based on the social-ecological model, self-determination theory, and habit theory described above. The mechanism labels and causal pathways were not rated separately by the expert panel.

### 2.9 Ethics

Ethics approval was issued by the Science and Technology Ethics Review Committee of Hengyang Normal University (Approval No. 2025lllw026), with an approval signature dated 21 December 2025. The expected project period of December 2025 to August 2026 is distinct from the approval date. Formal Stage 1 interviews began on 25 December 2025. One expert verbally accepted the invitation approximately 1 week before approval; this was preliminary recruitment contact. Written informed consent was obtained from all participating experts at their initial Stage 1 interview. Additional signed forms were obtained from 21 invitees in July 2026, before the initial submission on 8 August 2026: 13 members of the 16-person analysis set and eight noncompleters. These forms used the same terms as the original consent form. For the eight noncompleters, the additional signatures did not restore permission to use their study data; those data remain excluded, where contributed. The participant register identifies nine noncompleters, including two invitees who never entered Stage 1 and seven earlier-stage contributors whose data were withdrawn. None of the 16 included experts withdrew consent. Research records were de-identified for reporting.

## 3 Results

### 3.1 Panel participation and composition

The participant register records 25 experts approached, 23 completing Stage 1, 18 completing Stage 2, and 16 completing Stage 3. Two invitees did not enter Stage 1; five completed Stage 1 only; and two completed Stages 1 and 2 but not Stage 3. The register records withdrawal for all nine noncompleters and non-retention/non-use of study data where contributed. Eight noncompleters signed additional forms in July 2026, without restoring data-use permission or re-entering the analysis. Personal reasons for withdrawal were not recorded. None of the 16 included experts withdrew consent. Supplementary Table S8 distinguishes recruitment, participation, withdrawal, and the final analysis set.

### 3.2 Qualitative support for proposed domains and candidate development

The item-development sequence comprised 45 literature-derived provisional indicators and five interview-derived additions, forming a 50-item working pool, followed by consolidation to 34 candidates for Stage 2 rating. The additions were CS10 (load, safety, and recovery guidance), EAS10 (reliable extracurricular resource access), SPC9 (class- or signature-program culture building), IS11 (activity records and feedback), and IS12 (coordination across physical education, homeroom teaching, and mental health education). SPC9 did not meet the Stage 2 retention rule. Its exclusion appears in the Stage 3 decision summary but cannot be interpreted as a quantified individual-level consensus finding.

The 14-code analytical framework distinguished the five support themes (C05–C09) from conceptual, mechanism-related, and implementation codes. Comments on curricular support emphasized transferable skills, differentiated mastery, self-management, and safe participation. Extracurricular comments focused on stable time and place, continuity, accessible choice, and resource access. Peer comments addressed invitation, companionship, encouragement, belonging, and non-exclusion. Cultural comments concerned health-oriented meaning, participation for all, recognition of progress, and role models. Institutional comments addressed protected time, resources, teacher capacity, safety, quality feedback, and coordination. C10 and C11 captured cross-domain references to stable cues, repetition, autonomous motivation, and self-efficacy. Supplementary Table S7 provides author translations of de-identified original Chinese statements from the experts. Because the interview prompts were organized around the proposed domains, these findings document how the domains and indicators were represented in the interview material but do not provide independent psychometric or statistical validation of the five-domain structure.

### 3.3 Stage 2 item ratings

Of the 34 Stage 2 candidates, 33 met all three reported importance criteria. Across 544 importance scores (16 experts × 34 items), the mean was 4.436 (SD 0.536). Retained-item means ranged from 4.188 to 4.812, importance CVs from 0.084 to 0.115, and every retained indicator received a score of 4 or 5 from all experts. EAS5M had the highest importance mean (4.812, SD 0.403), followed by PS2M (4.750, SD 0.447). SPC9 was the sole non-retained candidate: importance mean 3.562 (SD 0.892), CV 0.250, and 68.8% of scores at 4 or 5.

Of 528 importance ratings for the 33 retained items, 244 were 5 (46.2%) and 284 were 4 (53.8%) in the available 16-person matrix. Raising the endorsement requirement from 80 to 90% or 100% therefore retains the same 33 candidates. With CV ≤ 0.20 and endorsement ≥80% unchanged, raising the mean cutoff to 4.25, 4.50, or 4.75 retains 31, 15, or 2 candidates, respectively (Supplementary Table S11). These are *post hoc* descriptions of the restricted analysis set, not validated alternative definitions of essential content.

Across the 544 feasibility ratings, the pooled mean was 3.947 (SD 0.740); item means ranged from 2.938 for SPC9 to 4.375 for CS2. Curricular support had the highest domain mean (4.250, SD 0.435), followed by extracurricular support (4.054, SD 0.815) and peer support (4.021, SD 0.821). Institutional support had the lowest domain mean (3.656, SD 0.477). No item had an SD of zero for importance, feasibility, or clarity.

Across all 544 clarity scores, the mean was 4.381 (SD 0.589), with item means from 2.750 for SPC9 to 4.750 for EAS9R. In the retained 33-item subset, pooled means were 4.462 (SD 0.499) for importance, 3.977 (SD 0.710) for feasibility, and 4.430 (SD 0.496) for clarity. Table 3 reports both the full 34-candidate selection set and this explicitly labelled retained subset; Supplementary Table S1 provides item-level results.

**Table 3:** Stage 2 ratings for all 34 candidates and the descriptive 33-item retained subset.

Analysis set	Domain	Items	Importance, mean (SD)	Feasibility, mean (SD)	Clarity, mean (SD)
A. All 34 Stage 2 candidates	Curricular support	7	4.509 (0.502)	4.250 (0.435)	4.393 (0.491)
A. All 34 Stage 2 candidates	Extracurricular physical activity support	7	4.518 (0.502)	4.054 (0.815)	4.554 (0.499)
A. All 34 Stage 2 candidates	Peer support	6	4.531 (0.502)	4.021 (0.821)	4.594 (0.494)
A. All 34 Stage 2 candidates	School physical activity culture	6	4.146 (0.598)	3.781 (0.943)	4.094 (0.847)
A. All 34 Stage 2 candidates	Institutional support	8	4.445 (0.499)	3.656 (0.477)	4.273 (0.447)
A. All 34 Stage 2 candidates	Overall	34	4.436 (0.536)	3.947 (0.740)	4.381 (0.589)
B. Retained 33-item subset	Curricular support	7	4.509 (0.502)	4.250 (0.435)	4.393 (0.491)
B. Retained 33-item subset	Extracurricular physical activity support	7	4.518 (0.502)	4.054 (0.815)	4.554 (0.499)
B. Retained 33-item subset	Peer support	6	4.531 (0.502)	4.021 (0.821)	4.594 (0.494)
B. Retained 33-item subset	School physical activity culture	5	4.263 (0.443)	3.950 (0.840)	4.362 (0.484)
B. Retained 33-item subset	Institutional support	8	4.445 (0.499)	3.656 (0.477)	4.273 (0.447)
B. Retained 33-item subset	Overall	33	4.462 (0.499)	3.977 (0.710)	4.430 (0.496)

Values combine expert-by-item ratings within each analysis set; SD is the sample standard deviation (n − 1). Panel A contains all 34 candidates used for the reported retention decision. Panel B excludes SPC9 and summarizes the unchanged Stage 2 ratings of the 33 retained items; it is not a Stage 3 rerating. Statistics were calculated from the 16-expert analytic dataset.

Pooled medians [Q1–Q3] across the 34 candidates were 4.0 [4.0–5.0] for importance, 4.0 [3.0–4.0] for feasibility, and 4.0 [4.0–5.0] for clarity. The full observed ranges were 2–5 for all three dimensions. Distribution-aware item summaries are reported in Supplementary Table S2.

### 3.4 Concordance

Tie-corrected Kendall’s W across 34 candidates was 0.115 for importance (*χ*^2^ = 60.863, df = 33, *p* = 0.002), 0.160 for feasibility (*χ*^2^ = 84.473, df = 33, *p* < 0.001), and 0.163 for clarity (*χ*^2^ = 86.284, df = 33, *p* < 0.001; Supplementary Table S9). These values indicate limited concordance in relative item rankings. In contrast, all 16 included experts rated each retained item as 4 or 5 for importance, indicating endorsement at that dichotomized threshold, not identical ratings or strong agreement on relative priorities. Statistical significance of W does not change its small magnitude and does not establish iterative consensus.

Feasibility ratings varied more than importance ratings across items and domains, particularly between curricular and institutional support. Nevertheless, Kendall’s W for feasibility remained low. The statistically significant result indicates non-random concordance in relative rankings but does not imply strong agreement or a generalizable ranking.

### 3.5 Exploratory importance-feasibility prioritization

Using the retained-subset grand means as crosshairs, 10 indicators fell in the priority-action quadrant: CS1, CS2, CS7M, CS8, CS10, EAS1, EAS10, EAS2M, PS7, and PS8M. Five indicators fell in the strategic-challenge quadrant because importance was at or above 4.462 while feasibility was below 3.977: EAS5M, PS1, PS2M, IS11, and IS12.

Ten indicators were classified as support and monitor: CS3, CS4R, EAS7R, EAS8R, EAS9R, PS4R, PS6R, SPC3M, SPC8R, and SPC7M. Eight were classified as foundational investment: SPC1R, SPC5R, IS1R, IS2R, IS3R, IS5R, IS6R, and IS8R. “Higher” and “lower” refer only to the sample-relative crosshairs; all retained indicators exceeded the reported absolute importance threshold.

Twenty-two of 33 retained indicators remained in their full-sample quadrant in all 16 leave-one-expert-out recalculations. The corresponding counts were 15/16 for CS1, 13/16 for EAS1, 12/16 for PS8M, 11/16 for EAS2M, 10/16 for PS2M, PS6R, SPC8R, and IS5R, 9/16 for EAS7R and IS8R, and 7/16 for EAS8R. Thus, 11 indicators changed quadrant under at least one omission. These counts are descriptive perturbation results, not confidence levels or evidence of external validation. The matrix should be used to pose local implementation questions, not to mandate a sequence of actions (see Figure 2).

**Figure 2:**
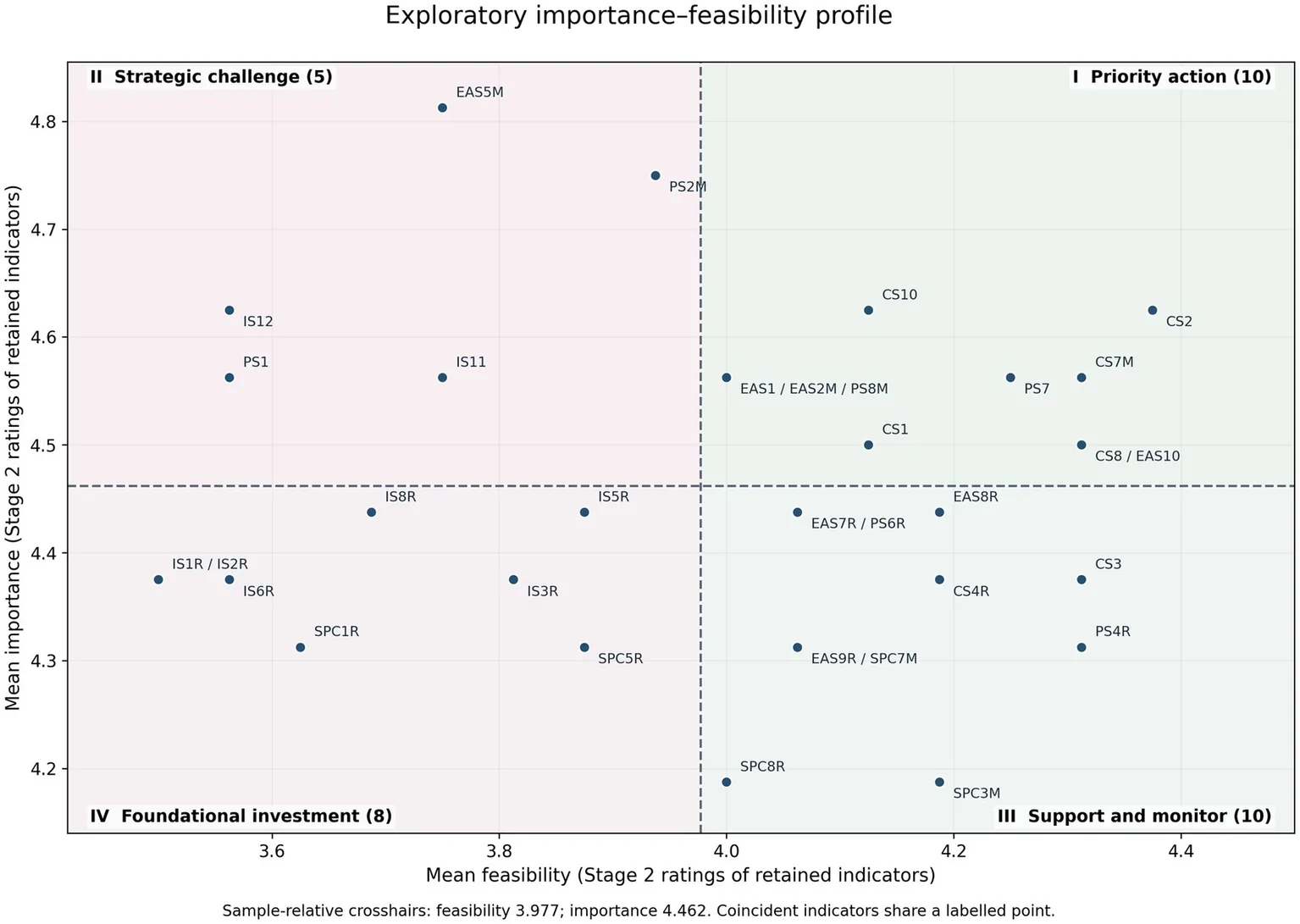
Exploratory, sample-relative importance–feasibility matrix for the 33 retained indicators using unchanged Stage 2 scores. Crosshairs are retained-subset grand means (importance 4.462; feasibility 3.977), not externally validated cutoffs. Quadrant names are descriptive labels, not tested implementation recommendations. Exact means determine assignments; coincident indicators share a labelled point. Eleven indicators changed quadrant in at least one leave-one-expert-out analysis.

### 3.6 Summary-level stage 3 decisions and the resulting framework

The Stage 3 summary records exclusion of SPC9, “Class- and signature-program physical activity culture building,” retention of the other 33 items without code or wording changes, and closure of the consultation. SPC9 was the only candidate failing the importance-based rule and had the lowest feasibility and clarity means. Sixteen participants were reported for this stage, but no individual confirmation distribution was available in the submitted analytic record. We therefore do not report a confirmation percentage, unanimity, increased consensus, or between-stage rating stability (Supplementary Table S4).

The final framework therefore contained 33 indicators: curricular support (7), extracurricular physical activity support (7), peer support (6), school physical activity culture (5), and institutional support (8) (Table 4). The numerical change from 34 to 33 reflects exclusion of SPC9, not a merger of two rated culture items.

**Table 4:** Final five-domain, 33-indicator school-based physical activity support framework.

Domain	Code	Final indicator	Proposed primary function (not tested)
Curricular support	CS1	Development of sustainable movement skills	Transferable capability
Curricular support	CS2	Integrated learning, practice, competition, and assessment	Coherent learning cycle
Curricular support	CS3	Differentiated teaching and successful mastery experiences	Competence and access
Curricular support	CS4R	Positive feedback and physical activity self-efficacy	Confidence and self-efficacy
Curricular support	CS7M	Integration of physical and mental health knowledge into physical education	Health knowledge internalization
Curricular support	CS8	Personal physical activity planning and self-management	Self-regulation and planning
Curricular support	CS10	Guidance on appropriate physical activity load, safety, and recovery	Safe, sustainable participation
Extracurricular physical activity support	EAS1	Scheduled extracurricular physical activity time	Stable time cue
Extracurricular physical activity support	EAS10	Reliable access to facilities and equipment	Stable resource access
Extracurricular physical activity support	EAS5M	Diverse, low-threshold physical activity options	Choice and accessible entry
Extracurricular physical activity support	EAS7R	Program continuity and periodized planning	Repetition and consolidation
Extracurricular physical activity support	EAS2M	Routine provision of active recess, after-school services, and sports clubs	Repeated school-day opportunity
Extracurricular physical activity support	EAS8R	Tiered activities with appropriate intensity	Graded, safe participation
Extracurricular physical activity support	EAS9R	Activity organization, guidance, and participation feedback	Organization and feedback
Peer support	PS1	Stable physical activity partners	Stable social cue
Peer support	PS2M	Peer invitation and companionship	Social initiation support
Peer support	PS4R	Peer encouragement and positive feedback	Positive reinforcement
Peer support	PS6R	Class- or club-based physical activity communities	Belonging and collective persistence
Peer support	PS7	Student leader facilitation	Role-modeling and facilitation
Peer support	PS8M	Cooperative, non-exclusionary peer interaction	Psychological safety and belonging
School physical activity culture	SPC1R	Health-promotion-oriented school physical activity philosophy	Shared health-promoting purpose
School physical activity culture	SPC3M	Culture of inclusive participation in physical activity	Inclusive norms and belonging
School physical activity culture	SPC8R	Recognition of sustained participation and progress	Recognition and reinforcement
School physical activity culture	SPC5R	Science-based communication about physical activity and physical and mental health	Evidence-based health meaning
School physical activity culture	SPC7M	Role-modeling, sport values, and thematic education	Identity, values, and role-modeling
Institutional support	IS1R	Protection of physical education and daily physical activity time	Organizational continuity
Institutional support	IS2R	Facility and equipment provision and resource optimization	Material opportunity
Institutional support	IS3R	Teacher guidance and professional development support	Staff capability
Institutional support	IS5R	Physical education quality monitoring and implementation improvement	Quality feedback loop
Institutional support	IS6R	Physical activity safety, risk assessment, and emergency management	Safe participation
Institutional support	IS11	Student physical activity recording and feedback mechanisms	Self-monitoring and feedback
Institutional support	IS12	Coordination among physical education, homeroom teaching, and mental health education	Integrated health support
Institutional support	IS8R	Family-school-community coordination, sport-medicine integration, and regional support	Cross-setting continuity and equity

M denotes merger and R denotes material revision before Stage 2 rating. The 33-item framework is not a validated scale. Primary functions are proposed research-team interpretations, not separately rated constructs or demonstrated mechanisms. Supplementary Table S10 clarifies overlapping functions without claiming that the rated wording has been revalidated.

### 3.7 Framework logic

Figure 3 presents the research team’s conceptual interpretation after item selection. The five proposed domains organize curricular learning and feedback, extracurricular opportunities and access, peer participation, shared school norms, and institutional arrangements. Their placement in the figure is not evidence that the domains are statistically distinct or that any particular school action changes student behavior.

**Figure 3:**
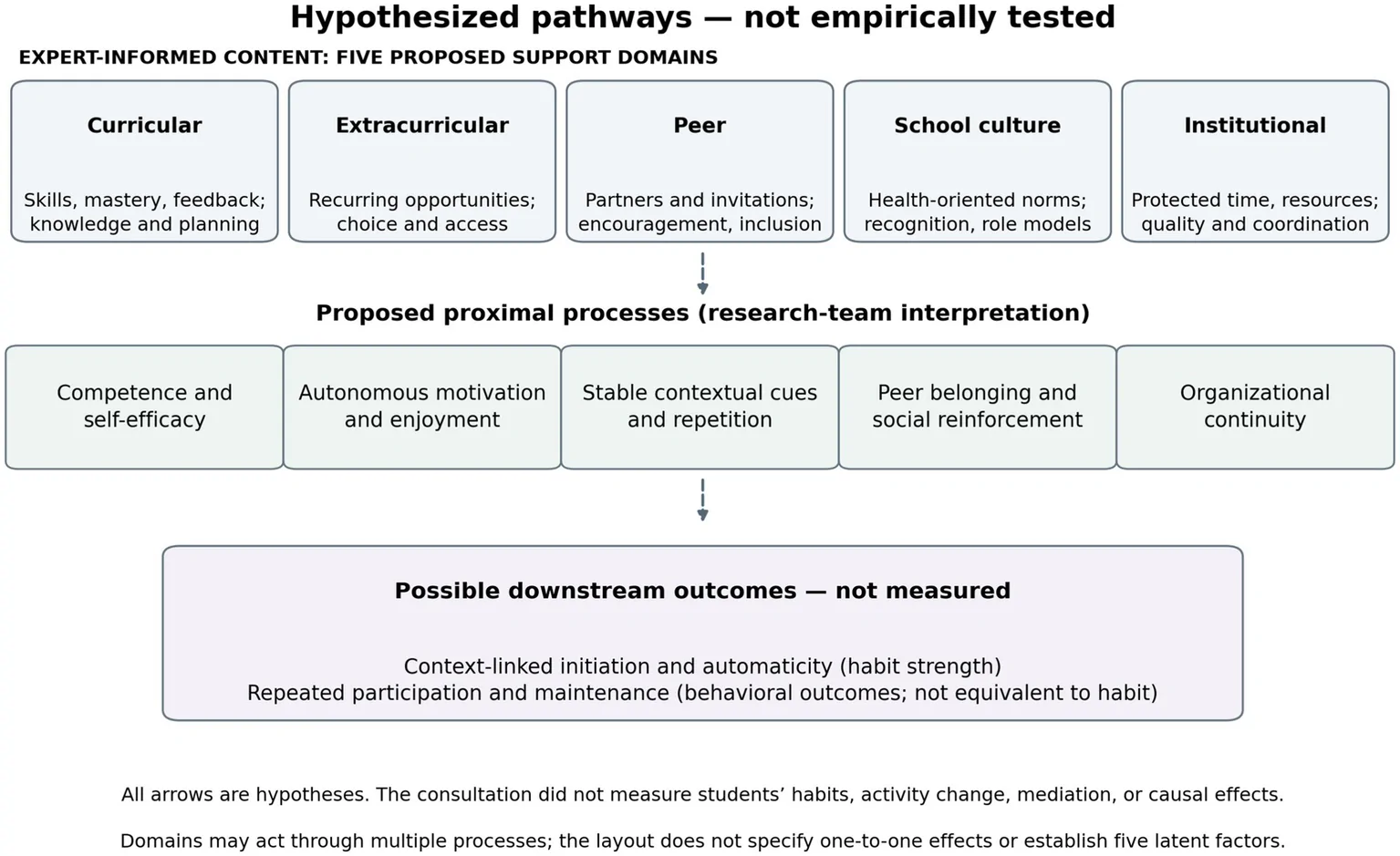
Hypothesized pathways linking school support conditions, proposed proximal processes, and possible downstream outcomes. All dashed arrows denote untested hypotheses. The expert exercise addressed framework content, not student habits, behavioral change, mediation, or causal effectiveness. Regular participation is not treated as evidence of automaticity. The mechanisms and pathways were organized by the research team and were not independently rated by the panel.

The domains are hypothesized to operate through competence and self-efficacy, autonomous motivation and enjoyment, stable contextual cues and repetition, peer belonging and social reinforcement, and organizational continuity. These mechanisms may contribute to context-linked initiation and automaticity and to repeated participation and maintenance, but those outcomes are not interchangeable. No student habit measure, longitudinal behavioral outcome, mediation model, or causal pathway was evaluated in this consultation. All arrows are explicitly hypothetical, and no one-to-one domain–mechanism correspondence is assumed.

## 4 Discussion

### 4.1 Principal findings

This three-stage, Delphi-informed consultation produced an expert-informed framework comprising five proposed domains and 33 indicators. The contribution is a specification of school conditions potentially relevant to habit support, not demonstration of habit formation. The reported working pool of 50 indicators was consolidated to 34 candidates; the single full quantitative rating stage identified 33 meeting the operational retention rule. The final-stage summary records closure of those decisions without providing an individual-level confirmation distribution.

Expert comments addressed each support domain, while stable cues, repetition, motivation, and self-efficacy appeared as cross-domain mechanisms rather than separate domains. Thirty-three of 34 candidates met the reported importance criteria; the reduction to 33 items resulted from exclusion of SPC9, not from a merger in Stage 3. Concordance in relative rankings was weak for importance, feasibility, and clarity. The exploratory matrix placed 10 indicators in the priority-action quadrant and five in the strategic-challenge quadrant, although 11 indicators changed quadrant in at least one leave-one-expert-out analysis. Feasibility ratings also showed variation within every domain.

The evidence supports content development and endorsement within the analyzed panel. It does not establish strong consensus, convergence across repeated quantitative rounds, a validated measurement instrument, or generalizability to other settings. Uniformly high importance ratings and weak relative-rank concordance are compatible: experts may consider many conditions important while ordering them differently. Dichotomizing 4 and 5 as endorsement also conceals distinctions in the original responses. Neither high means nor statistically significant W should be used to imply more agreement than these data show.

### 4.2 From activity opportunity to habit-supportive conditions

Much school physical activity work is organized around dose: more active lesson time, more breaks, more sessions, or more minutes of moderate-to-vigorous activity. Those goals remain essential. The present framework adds a different question: do students encounter opportunities in sufficiently stable, accessible, and rewarding conditions to repeat participation? Habit theory suggests that repetition in a stable context can strengthen cue–behavior associations, while reviews caution that habit and behavior influence each other and should not be conflated (26, 27, 29, 31, 34).

EAS5M, diverse low-threshold physical activity options, received the highest importance mean. Its value is not simply variety: accessible choice can reduce the skill, cost, and identity barriers that make participation difficult. When such options recur at predictable times and places, they may also provide stable cues. Yet repeated scheduling is not automatically habit-forming. If activities are compulsory, overly difficult, socially unsafe, or inconsistent, repetition may not support self-initiated maintenance. The framework therefore places accessible options alongside curricular competence, peer inclusion, feedback, and institutional continuity.

The proposed framework also avoids defining habit as high frequency alone. A student may be frequently active because participation is compulsory but stop when the external requirement disappears. Conversely, a student may develop a reliable initiation routine before achieving high overall volume. The Self-Report Habit Index was designed to capture repetition, automaticity, and identity-related features rather than frequency alone, although later synthesis has questioned whether all components should be combined indiscriminately (32, 33). Future validation should therefore measure physical activity volume and habit strength separately, ideally alongside contextual stability, enjoyment, competence, and autonomous motivation.

### 4.3 Interpreting the five domains

Curricular support provides the capability and self-regulation base. The final items address sustainable movement skills, an integrated learning-practice-competition-assessment cycle, differentiated mastery experiences, self-efficacy, physical and mental health knowledge, personal planning, and safe load and recovery guidance. This configuration is more precise than treating curriculum only as lesson time. Self-determination theory predicts that competence-supportive environments are more likely to foster autonomous engagement (23–25), while physical literacy scholarship links competence, confidence, motivation, knowledge, and continued participation (50, 51).

The curricular domain also connects learning with independent action. Students need not only exposure to activity but the ability to select an appropriate form, judge intensity and recovery, plan participation, and adapt it outside formal lessons. This is consistent with physical education’s public health role in preparing young people for continued activity (52–54) and with quality physical education guidance emphasizing inclusive learning and lifelong engagement (55).

Extracurricular support concerns recurring opportunities in which students may use curricular skills. EAS1 specifies a predictable scheduled time cue; EAS2M specifies routine delivery of recess, after-school, or club opportunities; and EAS7R specifies continuity across weeks or terms. IS1R, by contrast, concerns the organizational protection of time against displacement. Thus, an activity can be routinely offered without a stable cue or protected allocation, and a protected allocation may exist without reliable delivery. These distinctions concern design and implementation functions, not independently validated subconstructs (Supplementary Table S10) (56).

Peer support had the highest domain importance mean. Peer invitation and companionship were especially highly rated. The final peer domain distinguishes stable partners, invitation, encouragement, communities, student leader facilitation, and cooperative non-exclusionary interaction. These processes matter because adolescents often judge whether an activity is possible and socially safe through peers (35–37). A peer-led activity that increases visibility while preserving ridicule or exclusion would not satisfy this domain.

School physical activity culture concerns collective meaning rather than slogans. Its five retained indicators cover a health-promotion philosophy, inclusive participation, recognition of persistence and progress, science-based communication, and role-modeling or sport values. SPC9 was narrower, more dependent on local class or signature-program arrangements, and was not sufficiently endorsed or clear to remain as a separate indicator. Its exclusion does not remove class-level culture from consideration; those practices may serve as context-specific expressions of the broader retained culture indicators.

Institutional support is the enabling infrastructure. It includes protected time, facilities and resource optimization, teacher development, quality improvement, safety and emergency management, activity recording and feedback, cross-department coordination, and family-school-community or regional support. Its feasibility mean was the lowest of the five domains. IS11 and IS12 were strategic challenges because they combined above-crosshair importance with below-crosshair feasibility. The other six institutional indicators fell below both retained-subset crosshairs and were classified as foundational investments, reflecting scheduling, budget, access, supervision, and governance demands rather than low absolute importance.

Potentially overlapping indicators differ by the actor, action, temporal scale, and unit of implementation. CS4R concerns teacher-delivered mastery feedback; PS4R concerns interpersonal encouragement by peers; SPC8R concerns school-wide recognition norms; EAS9R concerns feedback within activity delivery; and IS11 concerns the system for longitudinal records and feedback. Self-efficacy and motivation are proposed learner processes, not substitutes for those observable supports. Similarly, PS7 concerns student leaders’ active facilitation, whereas SPC7M concerns role-model and values messages. Social reinforcement is a proposed cross-cutting mechanism, not an additional rated indicator. Supplementary Table S10 sets out these operational distinctions. They clarify intended meaning without demonstrating empirical discriminant validity or authorizing new wording to inherit earlier ratings.

### 4.4 Relationship to whole-school and Chinese socio-ecological frameworks

The framework aligns with health-promoting school and comprehensive school physical activity approaches in treating school health as an organizational task rather than the responsibility of the physical education teacher alone (16–20, 57). It differs in two ways. First, its organizing outcome is physical activity habit formation, which brings stable cues, repetition, initiation, and maintenance into the logic. Second, peers and culture are treated as explicit design domains rather than diffuse contextual influences.

The framework also occupies a different level from recent Chinese Delphi-based systems. Kong et al. (39) allocated responsibilities across school, family, community, and society and used analytic hierarchy process weights. Luo and Chen (40) evaluated government, school, family, and community support and applied their index system in a regional sample. Delphi–AHP methods have also been used to develop a physical literacy assessment framework for Chinese preschool children (58), while a separate Delphi study specified the content of a structured preschool movement program (59). Together, these studies illustrate the broader move from general promotion principles toward operational constructs. The present framework deliberately stays within the school and does not assign weights. Its institutional domain includes outward connections, but only where schools can organize or coordinate them. This narrower boundary allows more detail on physical education, after-school services, peer processes, inclusive culture, and internal governance.

The potential contribution is the operational organization of school-level supports around habit-relevant hypotheses, with separate descriptions of perceived importance and feasibility. The study does not introduce new behavioral theories or demonstrate superiority to existing frameworks. Its specifically Chinese arrangements—such as active recess, homeroom coordination, and regional teaching support—should be treated as context-dependent delivery forms. Transfer to another setting should preserve a plausible function while adapting delivery to local schedules, governance, resources, and cultural expectations, then test acceptability and outcomes. Cross-context applicability remains a proposition, not a finding.

### 4.5 Public health and implementation implications

The retained-subset matrix is an exploratory, sample-relative description. Its conventional labels—priority action, strategic challenge, support and monitor, and foundational investment—do not confer validated implementation status. For example, the relatively low feasibility of IS11 and IS12 could prompt investigation of data governance, staff time, and cross-department responsibilities; the matrix does not establish that either is an effective or optimal intervention. Local stakeholder priorities, dependencies, equity, and resources should determine which actions are tested.

Implementation should preserve the hypothesized mechanism. For example, adding an after-school activity is an opportunity intervention; maintaining the same time and place strengthens contextual stability; allowing meaningful choice and progression supports autonomy and competence; a buddy invitation process adds social reinforcement; and participation feedback can make progress visible. The behavior change wheel and COM-B model offer one way to map the 33 indicators to capability, opportunity, and motivation during intervention design (60). Adolescent evidence linking self-efficacy with adherence to activity recommendations supports treating confidence as a measurable process rather than a general aspiration (61). That mapping should be prospective rather than retrofitted after outcomes are known.

Feasibility ratings provided a more differentiated implementation profile than importance ratings. Curricular support had the highest feasibility mean and relatively limited dispersion, whereas ratings for extracurricular, peer, and cultural support were more variable and institutional support had the lowest mean. Implementation barriers should therefore be examined by domain rather than assuming that all highly important indicators require similar resources or organizational authority.

Equity requires particular attention. A “diverse menu” of activities does not guarantee equitable access if attractive options carry fees, require prior skill, or occur when transport is unavailable. Similarly, activity recording can support feedback but can also create surveillance concerns or reinforce performance comparison. Schools should use the minimum necessary data, define who can access records, avoid punitive use, and offer feedback that recognizes progress rather than only high performance.

Coordination between physical education and mental health education was retained as an institutional support, but the framework does not position physical activity as a substitute for clinical care. Physical activity can contribute to mental health promotion and may reduce depressive symptoms in some contexts (8, 9). Students with mental health conditions may still require assessment and evidence-based treatment. Coordination should therefore mean appropriate referral, trauma-sensitive and non-stigmatizing participation, shared health-promotion goals, and clear professional boundaries.

### 4.6 Using the importance-feasibility matrix to stage implementation

The matrix does not provide validated classes, a fixed ranking, or an implementation algorithm. Both crosshairs depend on this panel and on the 33-item retained set; removing an item or changing the expert sample can move the reference means. In leave-one-expert-out analysis, 11 items crossed a quadrant boundary at least once. The 22 unchanged assignments were stable only under these particular single-expert omissions. Neither set of results establishes stability under the inclusion of missing experts, other school contexts, alternative thresholds, or independent replication.

The priority-action quadrant contained 10 curricular, extracurricular, and peer indicators. CS2, EAS10, and PS7 illustrate actions that schools may organize through coherent teaching cycles, reliable resource access, and student facilitation. Their placement does not establish effectiveness; it identifies candidates for early implementation studies of reach, acceptability, fidelity, equity, and maintenance.

Five indicators fell in the strategic-challenge quadrant. EAS5M requires a genuinely diverse activity menu with accessible entry rather than nominal choice. PS1 and PS2M require durable, non-coercive peer structures that do not reproduce exclusion. IS11 requires decisions about purpose, privacy, access, and non-punitive use of activity data, whereas IS12 requires role clarity and referral boundaries across physical education, homeroom teaching, and mental health education.

The support-and-monitor quadrant contained 10 curricular, extracurricular, peer, and culture indicators. The foundational-investment quadrant contained two culture indicators and all six remaining institutional supports. This pattern should not make culture secondary or infrastructure dispensable. The crosshairs are comparative means within a highly endorsed retained set; local needs, dependencies, equity, and resources should take precedence over rigid quadrant assignment.

### 4.7 Strengths and limitations

The report separates item development, importance endorsement, relative-rank concordance, and exploratory prioritization. The submitted supplements already provide the 50-to-34 source-to-candidate mapping (Supplementary Table S3), final-stage decision summary (Supplementary Table S4), question-level interview summary (Supplementary Table S5), codebook (Supplementary Table S6), illustrative excerpts (Supplementary Table S7), participation flow (Supplementary Table S8), and concordance statistics (Supplementary Table S9). The separate ethics application and full interview guide add documented eligibility criteria and procedural information. These materials strengthen reporting transparency, but aggregate summaries and blank study forms do not establish participant-level compliance, recover missing ratings, or demonstrate iterative consensus. The updated participant register adds relevant experience, total working years, professional titles, invitation dates, the qualifications checker, and reported withdrawal status. The author-corrected dates also clarify the implementation period. These administrative details do not provide individual Stage 3 judgments or new rating data.

Several limitations qualify interpretation. First, the panel was small and recruited purposively through recommendations from institutional leaders and academic mentors. This route may favor established professional networks and shared perspectives; the extent of that selection effect was not measured. Reported relevant experience, total working years, professional titles, invitation dates and the qualifications checker now characterize the retained panel. These additions improve reporting but do not demonstrate representativeness; two affiliation fields still identify research areas rather than institutions. The municipal teaching-research and secondary-school management participants provide some practice-facing representation. Distinct participation by adolescents, parents, general classroom teachers, school health staff, and community users is not established. These 16 experts do not represent all invitees, professional groups, or school settings. Cognitive interviews, co-design, and implementation testing with these users are needed, including attention to accessibility and less-resourced schools.

Second, the five domains, domain-specific prompts, and unchanged codebook preceded the interviews, creating a risk of anchoring and confirmation within the proposed framework. Prespecification clarifies the analytical sequence but does not establish independent derivation of the domains. Separate pre-reconciliation coding files were unavailable, preventing an independent audit of the reported independent coding or estimation of inter-coder agreement. Future work should preserve dated coding exports and discrepant cases. Interview modes and records also differed: face-to-face interviews in Hunan were audio-recorded, whereas remote participation was documented through WeChat/telephone records. Confirmation that the published excerpts are original statements does not establish equivalent recording detail across every interview. Differences in probing, interaction, and documentation may influence the material; no mode-effect comparison was conducted. The supporting references now mapped in Supplementary Tables S12, S13 were assembled during revision. They clarify content relevance but do not recover the original item-extraction trail or independently validate the framework.

Third, the complete-case analysis excludes seven of 23 Stage 1 participants (30.4%) and two of 18 Stage 2 participants (11.1%). The ethics application documents a completion-based exclusion criterion, and the register records withdrawal and non-retention/non-use of prior data. Six of the seven excluded Stage 1 participants, including both excluded Stage 2 completers, signed additional forms in July without restoring data-use permission. The unavailable, withdrawn records cannot support full-stage reanalysis or comparisons of excluded versus retained ratings or interview content. Personal withdrawal reasons were not recorded, and we cannot determine whether withdrawal was related to views, availability, or perceived feasibility. The direction and magnitude of selection bias cannot be quantified. Leave-one-expert-out and threshold sensitivity analyses within the 16 retained cases do not remedy this limitation. No missing ratings or interview responses were reconstructed.

Fourth, retaining 33 of 34 candidates (97.1%) and observing only importance ratings of 4 or 5 for the retained set indicates restricted response variation and a possible ceiling effect. The operational thresholds selected broadly desirable content rather than establishing which indicators are essential, nonredundant, or sufficient. Increasing the endorsement percentage alone cannot discriminate between these retained items because all already meet 100% endorsement. *Post hoc* threshold sensitivity (Supplementary Table S11) illustrates dependence on the selection rule; it does not validate a replacement rule or justify choosing thresholds to obtain a preferred framework size. Best–worst scaling, forced prioritization, or resource-constrained tasks could be considered in a separately designed study.

Fifth, the actual consultation comprised open-ended expert interviews using a 32-question guide, one full quantitative rating stage, and final confirmation. The ethics application contains inconsistent summary descriptions: four open-ended questions in its process section and two quantitative consultation rounds in its methods section. These descriptions differ from the administered guide and the actual three-stage sequence reported here; the historical approval document has not been altered. The detailed process section and consent form describe final confirmation after rating. Individual Stage 3 confirmation distributions remain unavailable, so between-stage convergence, dissent at closure, and unanimity cannot be assessed. The pre-interview codebook in Supplementary Table S6 is distinct from the later question-level summary in Supplementary Table S5.

Sixth, the 33 indicators form a content framework, not a validated questionnaire, latent-variable model, or school-ranking index. Item boundaries in Supplementary Table S10 are interpretive clarifications and have not been independently evaluated. If a measurement instrument is developed, its unit of assessment, respondent groups, response options, scoring rules, and measurement model should first be specified. Factor analysis is relevant where reflective latent constructs are proposed, but five organizational domains should not automatically be assumed to be five reflective factors. Reliability, construct and predictive validity, responsiveness, and measurement invariance require appropriate empirical assessment. No total score, domain score, or cutoff is validated by the present expert exercise.

Finally, the proposed pathways from school support to motivation, repetition, automaticity, sustained physical activity, and health outcomes were developed after item finalization and were not independently rated or tested. Longitudinal and experimental research is needed to determine whether changes in framework domains precede changes in habit strength and whether habit mediates sustained behavior after active intervention support ends.

## 5 Conclusion

This study proposes an expert-informed, five-domain framework of 33 school-support indicators that may be relevant to adolescent physical activity habits. In the reported 16-person analysis set, 33 of 34 candidates met an operational importance-endorsement rule. That result should not be interpreted as strong inter-expert consensus or convergence through three quantitative rating rounds. Qualitative material supports the relevance and refinement of predefined domains but does not independently establish their structure.

The framework is a provisional content specification, not a validated measurement instrument or evidence that school support produces habit formation. The importance–feasibility matrix is exploratory and sample-relative, and all proposed pathways remain untested. Withdrawal-related loss of earlier-stage data limits the available analysis and cannot be corrected by the reported sensitivity analyses. Preservation of contemporaneous item-development records, stakeholder co-design, operational refinement, and prospective evaluation of measurement properties, implementation, and habit-related outcomes in diverse settings are needed.

## Data Availability

Aggregate rating summaries, the item-development mapping, codebook, question framework, and illustrative de-identified excerpts are provided in the supplementary materials. The full interview guide and consent-form template are also included in the submitted supporting package. No separate repository URL or accession identifier is reported. Access to retained de-identified individual ratings or interview materials may be considered on reasonable request where participant consent and institutional authorization permit. The consent form provides for destruction and non-use of data after withdrawal and retention of de-identified transcripts and aggregate ratings for two years after study completion. Requests should be directed to Shaojuan Hu, 2014010063@m.scnu.edu.cn. Withdrawn study data remain excluded from sharing and reanalysis. The additional July 2026 signatures did not restore data-use permission for the eight noncompleters. S8 summarizes administrative participation without publishing individual withdrawal dates or the professional profiles of withdrawn invitees.
