# Supplementary material for: An expert-informed framework of school conditions potentially supporting adolescent physical activity habits: a three-stage consultation in China
Source: Front Public Health. 2026 Oct 9;14:1962254. doi: 10.3389/fpubh.2026.1962254 (PMC13690933; doi:10.3389/fpubh.2026.1962254)
Supplement: Supplementary file 1 [file Data_Sheet_1.docx]

Supplementary consent form

Blank English consent template for publication. The approval date in Section VII has been corrected from 22 December 2025 to 21 December 2025 to match the approval signature date reported in Section 2.9 of the article. The consent and withdrawal terms are unchanged. This publication copy does not replace the signed consent records.

## **I. Basic Research Information**

**Research Title:** Developing a Five-Domain School-Based Physical Activity Support Framework for Adolescent Physical Activity Habit Formation: A Modified Delphi Study in China

**Research Institution:** College of Physical Education and Sports Science, Hengyang Normal University

**Principal Investigator:** Hu Shaojuan (College of Physical Education and Sports Science, Hengyang Normal University; 2014010063@m.scnu.edu.cn)

**Research Supervisors:** Xiong Jie, Li Liting, Han Dong, Li Xinyuan (College of Physical Education and Sports Science, Hengyang Normal University)

**Research Funding:** The Third Hunan Provincial Basic Education Teaching Reform Research Key Project (25JGZD0091); Hunan Provincial University Ideological and Political Education Research General Project (24SZ37); Key Scientific Research Project of the Hunan Provincial Department of Education (25A0483); Externally Commissioned Research Project Administered by Hengyang Normal University (HXKY2025028)

## **II. Research Purpose and Background**

This study aims to develop a **school-based physical activity support framework for adolescent habit formation**. The framework will systematically identify key school-level support factors that may facilitate adolescents in forming regular, autonomous, sustained, and stable physical activity habits across five domains: curricular support, extracurricular activity support, peer support, school physical activity culture, and institutional support.

As an expert panel member, you will participate in a **first-round semi-structured interview**. Your professional expertise and practical experience are invaluable for the preliminary construction of this framework.

## **III. Participation Content and Procedures**

If you agree to participate, the following procedures will be conducted:

**1. Interview Arrangements**

Interview method: In-person interview / telephone interview / WeChat voice call / video conference (whichever is most convenient for you)

Interview duration: Approximately 30–60 minutes

Interview content: Open-ended discussion concerning the five domains of school-based physical activity support (curricular support, extracurricular activity support, peer support, school physical activity culture, and institutional support)

**2. Subsequent Participation**

Approximately 4 weeks after the first-round interview, you will be invited to complete the second-round Delphi rating questionnaire

The second-round rating will take approximately 20–30 minutes

Approximately 3 weeks after the second round, you will be invited to participate in the third-round confirmation

The third-round confirmation will take approximately 10–15 minutes

**3. Total Research Duration**

The three rounds together will take approximately **60–105 minutes**, distributed across approximately 2–3 months.

## **IV. Privacy Protection and Data Use**

## **1. Audio Recording and Documentation**

## The interview will be audio-recorded (only with your consent). The recording will be used solely for accurate transcription and analysis.

## Audio recordings will be used for analysis purposes only and will be permanently deleted immediately upon completion of transcription and verification. They will not be used for any other purpose or retained beyond this stage.

## If you do not consent to audio recording, the researcher will take written notes only.

## **2. Data Confidentiality**

## All audio recordings and transcripts will be anonymized.

## Your personal information (name, affiliation, contact details) will not appear in any publicly available materials.

## When your views are cited in papers, reports, or academic conferences, only a random code (e.g., "Expert E05") will be used.

## **3. Data Storage**

## All raw data (audio recordings, transcripts, rating forms) will be stored on encrypted devices at the researcher's institution, with access restricted to core members of the research team.

## Interview audio recordings will be deleted immediately after transcription and verification, and will not enter the long-term storage phase.

## De-identified transcripts and aggregated rating data will be retained for 2 years after the completion of the study and then permanently deleted.

## **V. Voluntary Participation and Withdrawal Rights**

**1. Voluntary Principle**

Your participation is **entirely voluntary**.

You have the right to refuse to answer any question at any stage.

**2. Withdrawal Rights**

You have the right to **withdraw from this study at any time** without providing any reason.

Upon withdrawal, all data you have previously provided will be immediately destroyed and will not be used.

**3. No Adverse Consequences**

Your participation or withdrawal will not have any adverse effect on your academic status, career development, or relationship with the researcher's institution.

## **VI. Potential Risks and Benefits**

**1. Risks**

This study does not involve any experimental interventions, medications, or physiological tests.

The interview content concerns only professional opinions and academic judgments, and does not involve sensitive personal information.

The risk of discomfort from participating in this study is **minimal**, involving only the time required for normal academic discussion.

**2. Benefits**

You will contribute your professional expertise to the construction of a school-based physical activity support framework for adolescent habit formation.

You will have the opportunity to stay informed about the progress of this study.

Upon publication of the research results, you will receive acknowledgment (if you agree).

Upon request, the final published research paper will be provided to you.

## **VII. Ethics Review and Approval**

This study was reviewed and approved by the Science and Technology Ethics Review Committee of Hengyang Normal University (approval no. 2025lllw026; approved on 21 December 2025).

## **VIII. Informed Consent Statement**

### **Expert Statement**

I have carefully read and fully understood the entire content of this informed consent form. The research team has explained to me in detail the research purpose, participation content, privacy protection measures, risks and benefits, and my rights as a participant.

- I understand that my participation is voluntary and that I have the right to withdraw at any time.
- I understand that my responses will be used for academic publication, but my personal information will be strictly protected.
- I agree to participate in this study.

**Expert Signature:** ____________________

**Expert Name (Print):** ____________________

**Date:** ____________ Year ____________ Month ____________ Day

### **Interviewer Statement**

I have fully explained to the above expert the purpose, content, privacy protection measures, and participant rights of this study. I confirm that the expert understands the above information and has voluntarily agreed to participate.

**Interviewer Signature:** ____________________

**Name (Print):** Hu Shaojuan

**Date:** ____________ Year ____________ Month ____________ Day

## **IX. Contact Information**

If you have any questions, concerns, or wish to exercise your rights regarding this study, please contact:

**Principal Investigator:** Hu Shaojuan

**Email:** 2014010063@m.scnu.edu.cn

**Mailing Address:** College of Physical Education and Sports Science, Hengyang Normal University, No. 16 Henghua Road, Zhuhui District, Hengyang, Hunan, China (Postal Code: 421002)

**Research Supervisor:** Xiong Jie

If you have any complaints or concerns regarding the ethical issues involved in this study, please contact:

**Ethics Review Body:** Science and Technology Ethics Review Committee of Hengyang Normal University

## **X. Separate Consent for Audio Recording**

**I consent to the audio recording of the interview process, and I understand that the recording will be used solely for accurate transcription and analysis, and will be deleted after transcription.**

□ I consent to audio recording.

□ I do not consent to audio recording (the research team will take written notes only).

Note: If you consent to audio recording, please sign below.

**Expert Signature:** ____________________

**Date:** ____________ Year ____________ Month ____________ Day

**END**
