# Supplementary material for: An expert-informed framework of school conditions potentially supporting adolescent physical activity habits: a three-stage consultation in China
Source: Front Public Health. 2026 Oct 9;14:1962254. doi: 10.3389/fpubh.2026.1962254 (PMC13690933; doi:10.3389/fpubh.2026.1962254)
Supplement: Supplementary file 2 [file Supplementary_file_1.docx]

## Opening Remarks

Dear Expert Panel Member:

This study aims to develop a School-Based Physical Activity Support Framework for Adolescent Physical Activity Habit Formation. It seeks to clarify how schools can foster regular, autonomous, persistent, and stable physical activity habits among students through support related to the curriculum, extracurricular activities, peer interactions, school culture, and institutional arrangements. The Round 1 interview is designed to elicit your views on the framework domains, item content, conceptual wording, and applicability in school settings. Your feedback will be used to refine the initial item pool and develop the Round 2 Delphi rating questionnaire.

## Part I: Expert Background Information

1. What is your primary area of expertise?
2. School physical education / physical education curriculum and instruction
3. Sport and exercise psychology / physical activity behavior
4. Adolescent mental health / school psychology
5. Public health / health promotion
6. School administration / moral education / mental health education
7. Educational measurement / evaluation
8. Other: ______
2. How many years have you been engaged in relevant research or practice?
3. Have you been involved in any of the following activities?
11. School physical education curriculum reform
12. Promotion of physical activity among students
13. Development of health-promoting schools
14. Adolescent mental health education
15. Physical activity intervention research
16. Indicator-system development or Delphi research
17. Other: ______
4. How familiar are you with the topic of school-based physical activity support for adolescent physical activity habit formation?
   1 = Not at all familiar; 5 = Very familiar.

## Part II: Appraisal of Core Concepts

### Q1. How do you understand the concept of "school-based physical activity support"?

Prompts:

- Should it include the physical education curriculum?
- Should it include extracurricular activities?
- Should it include peer, cultural, and institutional support?
- Should it be distinguished from the broader concept of the "school physical activity environment"?

### Q2. Do you consider the concept of "physical activity habit formation" applicable to adolescents?

Please assess whether the following characteristics are appropriate:

- Regularity
- Autonomy
- Persistence
- Stability
- Automaticity
- Integration into daily life

### Q3. Do you believe that school-based physical activity support is associated with the promotion of adolescent mental health?

Prompts:

- Could physical activity habit formation serve as a mechanism through which physical activity promotes mental health?
- Does physical activity support in school settings have public health value?
- Is it appropriate to examine this topic within a health promotion framework?

## Part III: Appraisal of the Overarching Domains

Please evaluate whether the following five domains are appropriate core domains of a school-based physical activity support framework:

1. Curricular Support
2. Extracurricular Activity Support
3. Peer Support
4. School Physical Activity Culture
5. Institutional Support

### Q4. Do you consider these five domains comprehensive?

- Which domains should be retained?
- Which domains should be combined?
- Which domains should be divided into separate domains?
- Should any new domains be added?
- Which domain is the most important, and why?

### Q5. Is "School Physical Activity Culture" appropriate as a distinct domain?

If not, should it be incorporated into Institutional Support or Extracurricular Activity Support?

### Q6. What school-level provisions should be included in "Institutional Support"?

Prompts:

- Protected time
- Access to activity spaces and facilities
- Equipment
- Teacher guidance
- Safety management
- School policies
- After-school services
- Cross-departmental coordination

# Part IV: Domain-Specific Interview Questions

## A. Curricular Support

### Q7. How should physical education help students develop long-term physical activity habits?

Possible prompts:

- Motor skill acquisition
- Mastery experiences
- Autonomy and choice
- Health-related knowledge
- Personal physical activity planning
- Links between curricular and extracurricular physical activity
- Assessment approaches

### Q8. Please identify three to five of the most important curricular support indicators.

Examples:

- Physical education classes help students acquire movement skills that support lifelong participation.
- Physical education teachers provide positive feedback.
- Physical education classes guide students in developing personal physical activity plans.

## B. Extracurricular Activity Support

### Q9. How can extracurricular physical activity shift from merely "providing activities" to supporting habit formation?

Prompts:

- Regularly scheduled times
- Consistent locations
- Variety of activities
- Ease of participation
- Continuity of activities
- Low-barrier participation
- Support from student societies or sports clubs

### Q10. Which extracurricular activity arrangements are most conducive to the development of stable physical activity habits among students?

### Q11. Please identify three to five of the most important extracurricular activity support indicators.

## C. Peer Support

### Q12. How do peer relationships influence physical activity habit formation among adolescents?

Prompts:

- Invitations from peers
- Peer companionship
- Peer encouragement
- Physical activity partners
- Class-based physical activity groups
- Peer role models
- Sense of group belonging
- Non-competitive support

### Q13. Do class-based physical activity communities or groups facilitate habit formation? Why or why not?

### Q14. Please identify three to five of the most important peer support indicators.

## D. School Physical Activity Culture

### Q15. How can schools cultivate a culture in which "physical activity is part of everyday life"?

Prompts:

- Recognition of the value of physical activity
- Promotion of healthy lifestyles
- Participation beyond competitive sport
- An inclusive physical activity climate
- Encouragement through role models
- School-wide sports events
- Class-based challenges
- Student showcases and recognition

### Q16. How can schools avoid an excessive emphasis on competition that may cause students with lower physical competence to disengage?

### Q17. Please identify three to five of the most important school physical activity culture indicators.

## E. Institutional Support

### Q18. How should school policies and institutional arrangements safeguard students' sustained participation in physical activity?

Prompts:

- Protected time for physical activity
- Access to activity spaces and facilities
- Access to equipment
- Teacher guidance
- Safety management
- Cross-departmental coordination
- Participation and process records
- Feedback mechanisms
- School-family collaboration

### Q19. How should physical education teachers, homeroom teachers, school counselors or psychology teachers, and school administrators collaborate to support students' physical activity habit formation?

### Q20. Please identify three to five of the most important institutional support indicators.

# Part V: Mechanism-Mapping Questions

*Interviewer note: This section helps clarify the mechanisms by which the framework supports habit formation, rather than treating the framework as a simple list of indicators.*

### Q21. Which forms of school support are most conducive to establishing stable contextual cues?

Prompts:

- A consistent time
- A consistent location
- A consistent activity partner
- A consistent activity routine
- Physical activity logs or reminders

### Q22. Which forms of school support are most conducive to sustaining motivation to participate?

Prompts:

- Interest and enjoyment
- Autonomous choice
- Mastery experiences
- Teacher encouragement
- Peer companionship
- Recognition of the value of physical activity

### Q23. Which forms of school support are most conducive to strengthening physical activity self-efficacy?

Prompts:

- Skill mastery
- Progressive challenges
- Positive feedback
- Attainable goals
- Teacher guidance

### Q24. Which forms of school support are most conducive to developing automaticity in physical activity habits?

Prompts:

- Stable contexts
- Repeated participation
- Low-barrier activities
- Integration into daily routines
- Participation with peers

# Part VI: School Application and Health Promotion

### Q25. What value could this framework have for school-based health promotion practice?

Prompts:

- Promotion of physical activity among students
- Development of health-promoting schools
- Promotion of adolescent mental health
- After-school services
- Physical education curriculum reform

### Q26. If this framework were implemented in secondary schools, which indicators would be easiest to implement, and which would be most difficult?

### Q27. Which indicators are more appropriately assessed by students, and which are more appropriately assessed by teachers or school administrators?

### Q28. Which indicators could be operationalized as items in a subsequent scale, and which would be more suitable as components of an intervention program?

# Part VII: Prioritization and Additional Comments

### Q29. From the full set of indicators, please select the 10 that should be given the highest priority for retention.

### Q30. Please identify the indicators that are least clear, least feasible, or least necessary.

### Q31. What important support factors, if any, are still missing from this framework?

### Q32. Which of the following titles would you recommend for the final framework?

1. School-Based Physical Activity Support Framework
2. School-Based Support Framework for Adolescent Physical Activity Habit Formation
3. Health-Promoting School Physical Activity Support Framework
4. School Physical Activity Habit Formation Support Framework
5. Other: ______
