# Supplementary material for: An expert-informed framework of school conditions potentially supporting adolescent physical activity habits: a three-stage consultation in China
Source: Front Public Health. 2026 Oct 9;14:1962254. doi: 10.3389/fpubh.2026.1962254 (PMC13690933; doi:10.3389/fpubh.2026.1962254)
Supplement: Supplementary file 3 [file Supplementary_file_2.docx]

## 访谈开场说明

尊敬的专家：

本研究拟构建 **青少年身体活动习惯形成的学校身体活动支持框架**，旨在明确学校如何通过课程、课外活动、同伴互动、校园文化和制度保障等支持因素，促进学生形成规律、自主、持续、稳定的身体活动习惯。第一轮访谈主要听取您对框架维度、指标内容、概念表述和学校适用性的意见。您的建议将用于修订初始指标池，并形成第二轮 Delphi 评分问卷。

## 第一部分：专家背景信息

1. 您的主要专业领域是什么？
2. 学校体育/体育课程与教学
3. 运动心理/运动行为
4. 青少年心理健康/学校心理
5. 公共卫生/健康促进
6. 学校管理/德育/心理健康教育
7. 教育测量/评价
8. 其他
2. 您从事相关研究或实践的年限是多少？
3. 您是否参与过以下工作？
11. 学校体育课程改革
12. 学生身体活动促进
13. 健康学校建设
14. 青少年心理健康教育
15. 运动干预研究
16. 指标体系或 Delphi 研究
17. 其他
4. 您对“学校身体活动支持促进青少年身体活动习惯形成”这一主题的熟悉程度如何？
   1 = 很不熟悉，5 = 非常熟悉。

## 第二部分：核心概念判断

### Q1. 您如何理解 “School-Based Physical Activity Support / 学校身体活动支持”？

提示：

- 它是否应包括体育课程？
- 是否应包括课外活动？
- 是否应包括同伴、文化和制度支持？
- 是否需要区别于一般 “school physical activity environment”？

### Q2. 您认为 “Physical Activity Habit Formation / 身体活动习惯形成” 对青少年是否适用？

请判断以下特征是否合理：

- 规律性 regularity；
- 自主性 autonomy；
- 持续性 persistence；
- 稳定性 stability；
- 自动化 automaticity；
- 日常生活整合 daily-life integration。

### Q3. 您认为学校身体活动支持与青少年心理健康促进之间是否存在关联？

提示：

- 运动习惯是否可能成为身体活动促进心理健康的中间机制？
- 学校场景中的身体活动支持是否具有公共健康价值？
- 是否适合放在 health promotion 框架中讨论？

## 第三部分：一级维度判断

请您评价以下五个一级维度是否适合作为学校身体活动支持框架的核心维度：

1. Curricular Support 课程支持
2. Extracurricular Activity Support 课外活动支持
3. Peer Support 同伴支持
4. School Physical Activity Culture 校园身体活动文化
5. Institutional Support 制度支持

### Q4. 您认为这五个维度是否完整？

- 哪些维度应保留？
- 哪些维度需要合并？
- 哪些维度需要拆分？
- 是否需要增加新的维度？
- 哪个维度最关键？为什么？

### Q5. 您认为 “School Physical Activity Culture” 是否适合作为独立维度？

如果不适合，是否应归入制度支持或活动支持？

### Q6. 您认为 “Institutional Support” 应包含哪些学校层面的保障？

提示：

- 时间；
- 场地；
- 器材；
- 教师指导；
- 安全管理；
- 学校政策；
- 课后服务；
- 多部门协同。

# 十二、五个维度的具体访谈问题

## A. 课程支持 Curricular Support

### Q7. 体育课程应如何促进学生形成长期身体活动习惯？

可提示专家从以下方面回答：

- 运动技能学习；
- 成功体验；
- 自主选择；
- 健康知识；
- 运动计划；
- 课内外衔接；
- 评价方式。

### Q8. 请您列出 3—5 个最重要的课程支持指标。

例如：

- 体育课帮助学生掌握可长期参与的运动技能；
- 体育教师提供积极反馈；
- 体育课引导学生制定个人运动计划。

## B. 课外活动支持 Extracurricular Activity Support

### Q9. 学校课外身体活动应如何从“活动供给”转向“习惯支持”？

提示：

- 固定时间；
- 固定地点；
- 项目多样性；
- 参与便利性；
- 活动连续性；
- 低门槛参与；
- 社团或俱乐部支持。

### Q10. 哪些课外活动安排最有利于学生形成稳定身体活动习惯？

### Q11. 请您列出 3—5 个最重要的课外活动支持指标。

## C. 同伴支持 Peer Support

### Q12. 同伴关系如何影响青少年身体活动习惯形成？

提示：

- 同伴邀请；
- 同伴陪伴；
- 同伴鼓励；
- 运动伙伴；
- 班级运动小组；
- 同伴榜样；
- 群体归属；
- 非竞争性支持。

### Q13. 班级运动共同体或运动小组是否有助于习惯形成？为什么？

### Q14. 请您列出 3—5 个最重要的同伴支持指标。

## D. 校园身体活动文化 School Physical Activity Culture

### Q15. 学校应如何营造“身体活动是日常生活方式”的文化氛围？

提示：

- 运动价值认同；
- 健康生活倡导；
- 非竞技化参与；
- 包容性运动氛围；
- 榜样激励；
- 校园赛事；
- 班级挑战；
- 学生展示与认可。

### Q16. 如何避免校园运动文化过度竞技化，导致低运动能力学生退出？

### Q17. 请您列出 3—5 个最重要的校园身体活动文化支持指标。

## E. 制度支持 Institutional Support

### Q18. 学校制度应如何保障学生持续参与身体活动？

提示：

- 时间保障；
- 场地保障；
- 器材保障；
- 教师指导；
- 安全管理；
- 部门协同；
- 过程记录；
- 反馈机制；
- 家校协同。

### Q19. 体育教师、班主任、心理教师和学校管理者应如何协同支持学生身体活动习惯形成？

### Q20. 请您列出 3—5 个最重要的制度支持指标。

# 十三、机制映射问题

这一部分很重要，可以体现论文不是简单指标罗列，而是面向 habit formation 的 public health framework。

### Q21. 哪些学校支持因素最有助于形成稳定情境线索？

提示：

- 固定时间；
- 固定地点；
- 固定伙伴；
- 固定活动程序；
- 运动日志或提醒。

### Q22. 哪些学校支持因素最有助于增强持续参与动力？

提示：

- 兴趣；
- 自主选择；
- 成功体验；
- 教师鼓励；
- 同伴陪伴；
- 价值认同。

### Q23. 哪些学校支持因素最有助于增强运动自我效能？

提示：

- 技能掌握；
- 逐步挑战；
- 正向反馈；
- 可达成目标；
- 教师指导。

### Q24. 哪些学校支持因素最有助于促进身体活动习惯自动化？

提示：

- 稳定场景；
- 重复参与；
- 低门槛活动；
- 日常化安排；
- 同伴共同参与。

# 十四、学校应用与健康促进问题

### Q25. 您认为该框架对学校健康促进实践有什么价值？

提示：

- 学生身体活动促进；
- 健康学校建设；
- 青少年心理健康促进；
- 课后服务；
- 体育课程改革。

### Q26. 如果该框架用于中学实践，哪些指标最容易实施？哪些指标最难实施？

### Q27. 哪些指标更适合由学生评价？哪些指标更适合由教师或学校管理者评价？

### Q28. 哪些指标可以转化为后续量表题项？哪些更适合转化为干预方案内容？

# 十五、优先级与补充意见

### Q29. 请您从所有指标中选出最应优先保留的 10 个指标。

### Q30. 请您指出最不清晰、最难操作或最不必要的指标。

### Q31. 您认为本框架还遗漏了哪些重要支持因素？

### Q32. 您建议本框架最终命名为哪一种？

1. School-Based Physical Activity Support Framework
2. School-Based Support Framework for Adolescent Physical Activity Habit Formation
3. Health-Promoting School Physical Activity Support Framework
4. School Physical Activity Habit Formation Support Framework
5. 其他：____
